# Feed Curves for Controlling Ring Rolling Stability in Large-Scale Flat Ring Rolling Process

**DOI:** 10.3390/ma16093383

**Published:** 2023-04-26

**Authors:** Dan Xie, Qiu-yue Ouyang, Luo-yu He, Wu-jiao Xu

**Affiliations:** 1 College of Material Science and Engineering, Chongqing University, Chongqing 400044, China; 2 Chongqing Key Laboratory of Advanced Mold Intelligent Manufacturing, Chongqing 400044, China

**Keywords:** large-scale ring rolling, coordinated feed curve, ring rolling stability, finite element method

## Abstract

Due to the large wall thickness difference and serious instability in the large-scale ring rolling process, most studies on the feed curve are not suitable for a large-scale ring. The production cost of the large-scale ring is high, and if plastic instability occurs, it will cause a great waste of resources. Therefore, in this study, a staged feed strategy based on the evolution of ring instability is proposed with the objective of controlling the rolling stability of a large-scale ring. Firstly, based on the law of rolling instability evolution, the rolling stage during the rolling process is divided. Secondly, the coordination of all rolling stages is proposed as a factor to design the feed curve. The feed scheme is determined using the central composite design (CCD) method, and then the established mathematical model is applied to obtain the radial feed curves of a large-scale flat ring with a 5 m diameter for different schemes. Next, the designed feed curve was submitted to finite element method (FEM) simulation. According to the FE simulation results, a rolling map for controlling roundness error, eccentricity and vibration is established. Finally, the feed curve in the stable region is input to the FE simulation and the production trial to obtain the results of roundness error, eccentricity and vibration. A comparison of the simulation and production trial results shows that they are in good agreement, which proves the reliability of the feed curve designed based on the stable rolling region in the roll map. Moreover, the machining amount for both the simulation and production trial is below the maximum machined value.

## 1. Introduction

**Figure 1:**
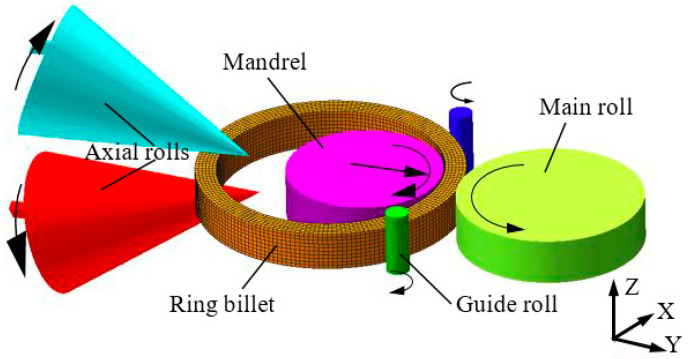
Ring rolling process.

Ring rolling is a special forming process that reduces the wall thickness and enlarges the diameter of a ring by controlling the radial and axial rolls. As an advanced forming technology, ring rolling has many advantages, such as good surface quality, good streamline and a high material utilization rate [1,2]. It is widely used in industrial fields such as aerospace, nuclear and wind energy. During the ring rolling process shown in Figure 1, the roles of different rolls are as follows [3,4,5]. The main roll acts as the driving roll to rotate the ring, while the mandrel feeds toward the main roll to make the ring wall thickness thinner and the ring diameter larger, and the gap between the main roll and mandrel is called the radial deformation zone. The upper axial roll feeds in the direction of the lower axial roll to control the axial height of the ring. The left and right guide rolls are close to both sides of the ring in the ring rolling process, whose function is to prevent the ring from swinging and control the stability of ring rolling.

In a large-scale ring rolling process, due to the characteristics of large ring diameter, large rolling forces and long rolling time, it is very critical and complicated to match the feed of working rolls and ring diameter growth dynamically [6,7,8]. Guo et al. [9] developed a mathematical model to predict ring diameter expansion and proposed that the constant diameter growth speed alleviates dynamic instability during rolling. However, it was not taken into account that the continuous feed of work rolls caused the variation of ring thickness along the circumference, which essentially affected the precision of this model in large-scale ring rolling.

Another characteristic of large-scale ring rolling is that rolling instability is prone to occur. The feed rate is the main factor to control rolling stability, so it is significant to study the feed curve. Lee et al. [10] obtained the feasible feed curve to reduce ring spread. Zhu et al. [11] analyzed the effects of radial–axial feed curve coordinates on the ring rolling process. Xu et al. [12] designed the feed interval considering multiple constraints by the analysis of FEM. Berti et al. [13] designed the ring feeding strategy by considering the thickness and height variation of the ring during rolling based on the shape of the cut surface at different locations of the ring. Liang et al. [14] described the ring diameter growth behavior at different axial positions and then established a matching relationship between the ring growth rate and the mandrel feed rate. They all control the ring rolling process based on the relationship between the ring growth speed and the feed rate, but coordination of different rolling stages in the ring rolling process is not considered. Liang et al. [15] studied a feeding strategy model for the staged growth of a ring but did not investigate the effect of plastic destabilization evolution of a large-scale ring.

With the development of computer technology and the finite element method (FEM), FEM simulation becomes the main tool to address ring rolling issues [16,17,18]. Hu et al. [19] studied the ring rolling process by using the elastic–plastic FEM and a hybrid FE mesh model. This method can improve the simulation accuracy and speed significantly. Han et al. [20] reported a FEM simulation and experimental research on cylindrical ring rolling. Hu et al. [21] used the FEM to study the effect of the roll cavity on the forming of ring parts. Luca et al. [22] simulated the strain field of the ring by FEM. Hua et al. [23] obtained an appropriate adjustment coefficient to determine the guiding force by FE simulation. Cleaver et al. [24] revealed the curvature development in ring rolling by means of FEM simulation and experiment. Lv et al. [25] investigated the effect of different rolling processes on the residual stresses in ring parts by FEM.

Aiming at the problem of poor stability during the rolling of a large-scale ring, this paper proposes a new design idea for feed strategy. At first, the growth of ring is divided into four stages according to the destabilization evolution law of ring, and then the optimized feed curve is finally obtained with the goal of coordinating the four stages. 

This paper is organized as follows. In Section 2, by analysis of the law of rolling instability evolution, the relationship between feed rate and rolling instability is determined, and the rolling stages during the rolling process are divided into the initial rolling stage, rapid rolling stage, steady rolling stage and slow rolling stage. The radial feed curves for controlling rolling stability are designed in Section 3. The first step is to determine the radial feed curve for different rolling stages. Then, based on the percentage range of wall thickness reduction in the rapid and slow rolling stages, the feed scheme is determined using the central composite design (CCD) method, and then the established mathematical model is applied to obtain the radial feed curves of a large-scale flat ring with a 5 m diameter for different schemes. Here, a three-dimensional thermal–mechanical coupling FE model of ring rolling based on the SIMUFACT platform is established. Finally, a rolling map for the coordinated rolling stage is built to obtain the stable rolling region by FEM simulation results of roundness error, eccentricity and vibration. Section 4 verifies whether the feed curve designed based on the stable rolling region by numerical simulation and production trial can control the rolling stability and geometric accuracy. The conclusions are summarized in Section 5.

## 2. Analysis of Ring Rolling Stability

Due to serious instability in the large-scale ring rolling process, defects such as large roundness error, eccentricity and vibration of the rolled ring easily occur in the rolling process. Therefore, the formation mechanism of roundness error, eccentricity and vibration needs to be analyzed in order to design a feed curve that can control the rolling stability of the rolled ring.

### 2.1. Roundness Error Analysis

**Figure 2:**
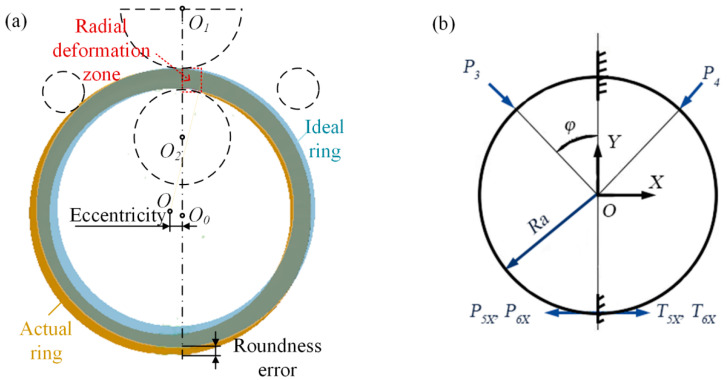
(**a**) Formation mechanism of roundness error and eccentricity and (**b**) mechanical model of ring.

Roundness error refers to the amount of variation of the actual circle measured in relation to the ideal circle, and thus this variation reflects the difference in geometry between the actual ring and the ideal ring. When comparing the geometry of a ring with a large roundness error with that of an ideal ring, as shown in Figure 2a, the difference is mainly in the loss of roundness in the direction of the entrance to the radial deformation zone, which is due to plastic instability in the radial deformation zone, i.e., the bending moment of the cross-section in the radial deformation zone is greater than the ultimate bending moment. Therefore, the reason for the large roundness error of the ring is that the bending moment M in the radial deformation zone is greater than the ultimate bending moment.

The large-scale ring is regarded as a circular curved beam with the gap between the main roll and mandrel as fixed supports and the gap between the upper and lower cone roll as smooth contact surface constraints. According to the mechanical model of the ring under the double guide rolls stage as shown in Figure 1b, the coordinate system is established with the circular center of the ring *O* as the reference point, and the M in the cross-section of the radial deformation area is calculated as follows.

(1) $$M=P_{3}R_{a}\sin\varphi -P_{4}R_{a}\sin\varphi +\mu P_{5}D_{a}+\mu P_{6}D_{a}-P_{5X}D_{a}-P_{6X}D_{a}$$

where *R_a_* and *D_a_* are the average radius and average diameter of the ring, respectively. $P_{3}$, $P_{4}$, $P_{5X}$ and $P_{6X}$ represent the rolling forces of the rolls on the ring. φ is the position angle of the guide roll, and  μ is the friction coefficient between the roll and the ring. 

From Equation (1), M is determined by the rolling force and ring size; however, the rolling force and ring size are determined by the feed curve. Therefore, the roundness error of the rolled ring can be controlled by optimizing the feed curve.

### 2.2. Eccentricity Analysis

For the ideal rolled ring, the center of the ideal ring *O*_0_ is always located on the line between the center of the mandrel *O*_2_ and the center of the main roll *O*_1_ during the rolling process. However, when the M on the curved beam cross-section is greater than the ultimate plastic bending moment, the cross-section becomes a plastic hinge [24], and at the same time, the central axes at the two ends of the plastic hinge rotate relatively. The rotation of the neutral axis causes the curvature of the ring in the circumferential direction to change, and this change in curvature causes the center of the ring *O* to be shifted, as shown in Figure 1a. Based on the above analysis, the reason for the eccentricity of the ring is also that the bending moment in the radial deformation zone is greater than the ultimate bending moment. Similar to the roundness error of the ring, the feed curve affects M by determining the rolling force and the size of the ring.

### 2.3. Vibration Analysis

Due to the nonlinear, non-stationary, asymmetric, and multipass rolling characteristics of the ring rolling process itself, vibration phenomena occur in the main roll, mandrel, guide roll and transmission and power components of the ring rolling machine, resulting in the radial rolling process of the ring with the deviation vibration of the mandrel. To avoid ring vibration, the guide rolls on the left and right sides of the ring are in contact with the outer surface of the ring. As the guide rolls move in real time through the outer diameter of the ring, the loss of circularity causes the outer diameter of the ring at the left and right guide rolls to be different, thus making it impossible for the left and right guide rolls to make contact with the ring to avoid ring vibration. Hence, the ring rolling process is accompanied by a deflection of the ring around the center of the mandrel during the non-guided roll control stage. This is due to the deflecting moment $M_{r}$ of the ring, which causes the ring to move in the radial plane around the center of the mandrel towards the right guide side. 

**Figure 3:**
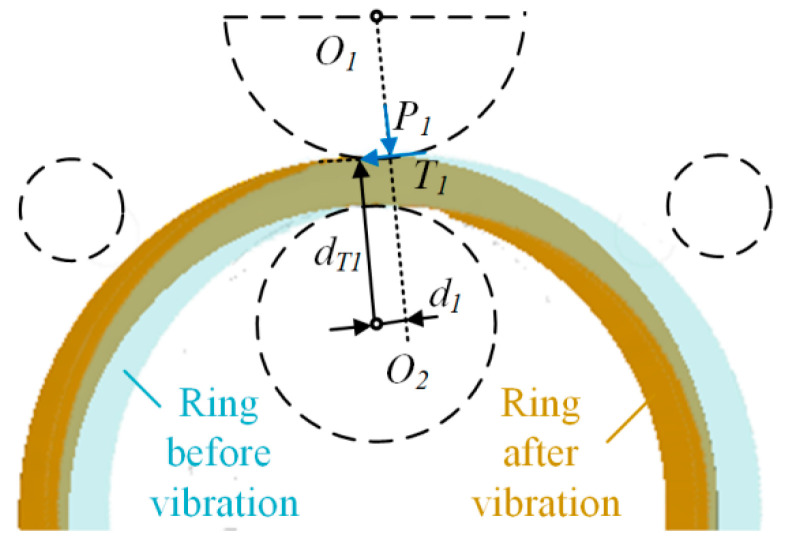
Ring vibration mechanical model.

Using the center of the mandrel *O*_2_ as the point of action, the torque on the ring is analyzed and calculated as shown in Figure 3. In order to simplify the calculation, it is approximated that the point of action of the combined force of the rolls on the ring is located at the midpoint of the contact arc, and the deflection moment of the ring is calculated as follows.

(2) $$M_{r}=T_{1}d_{T1}-P_{1}d_{1}$$

where $T_{1}$ is the friction force between the main roll and the ring, which can be expressed by $T_{1}=\mu P_{1}$ $d_{T1}$ is the arm length from the friction force to the mandrel center and $d_{1}$ is the arm length from the rolling force of the main roll on the ring to the mandrel center, which both vary with the wall thickness of the ring.

Equation (2) shows that $M_{r}$ is determined by the rolling force and the wall thickness of the ring. Combined with roundness error and eccentricity analysis, optimizing the feed curve can control defects such as roundness error, eccentricity and vibration due to ring rolling instability. It is worth noting that in large-scale ring rolling production, axial feeds are mainly used to eliminate end-fishtailing, while radial feeds play a role in thinning ring wall thickness and expanding the ring outer diameter. Therefore, the object of this study is the radial feed curve. 

### 2.4. Division of Rolling Stages Based on Rolling Stability

Combined with the formation mechanism of roundness error, eccentricity and vibration, rolling force and ring size are the main factors affecting rolling stability. The rolling force is positively correlated with the radial feed rate, so the rolling force can change with the radial feed curve during rolling. However, regardless of the radial feed curve, the ring size is constantly increasing, and thus the feed curve does not effectively reduce ring rolling instability by regulating the ring size. The rolling instability is significantly higher in the late stage of rolling compared to the early and middle rolling stages. Thereby, the radial feed curve should be different in the early, middle and late stages of rolling. The smaller size of the ring at the early stage of rolling makes for a smaller M and $M_{r}$ and a lower degree of ring instability; thus, the outer diameter of the ring should be increased rapidly at this stage. With the increase in the outer diameter of the ring, rolling instability increases, and at this time, the roundness of the ring is insufficient such that its guide roll cannot control the ring rolling stability, so the middle stage of rolling should take a constant ring growth speed to avoid the collision between ring and rolls. In order to alleviate the substantial increase in rolling instability in the late stage of rolling, a slow rolling strategy is adopted in the late stage of rolling. In addition, a low feed rate is required for the initial rolling to allow the ring to be bitten into the gap between the main roll and the mandrel and to rotate continuously. Hence, based on the law of rolling instability evolution, the different rolling stages during the entire rolling process are sequentially divided: initial rolling stage, rapid rolling stage, steady rolling stage and slow rolling stage.

## 3. Radial Feed Curve Design 

### 3.1. Radial Feed Curve for Different Rolling Stages

Radial feed curves are usually available in the form of increasing rate, constant rate and decreasing rate. Based on the radial feed rate analysis for the different rolling stages, the initial rolling stage requires a low feed rate, while the rapid rolling stage needs a relatively high feed rate for a rapid increase in the ring outer diameter. The feed curve for the steady rolling stage is calculated from a constant ring growth speed and is often a form of deceleration. After that, the radial feed rate is gradually reduced in the slow rolling stage to improve a substantial increase in the ring rolling instability. In order to match these stages, the radial feed rate in the initial stage is increased uniformly, and the feed curve in the rapid rolling stage is set to a uniform rate. The radial feed rate is uniformly reduced in the slow rolling stage to improve a substantial increase in the ring rolling instability. In addition to the form of the feed curve, it is also necessary to determine the rate values for the different rolling stages, which are obtained based on the relationship between the growth speed of the ring outer diameter and the radial feed rate.

According to the principle of ring volume invariance and the neglect of axial spread, formulas are expressed as follows:

(3) $$\frac{\pi }{4}\left(D^{2}-d^{2}\right)=\frac{\pi }{4}\left(D_{0}{}^{2}-d_{0}{}^{2}\right)$$

where $D_{0}$ is the initial outer diameter of the ring billet. $d_{0}$ is the initial inner diameter of the ring billet. D and d represent the instantaneous outer diameter and inner diameter of the ring during the rolling process, respectively. By taking the derivation of D with respect to rolling time t, according to the literature [11], it can be obtained as follows:

(4) $$v_{D}=\left[1-\frac{\left(D_{0}+d_{0}\right)H_{0}}{2H^{2}}\right]\frac{\partial H}{\partial t}$$

where $H_{0}$ is the initial wall thickness and H represents the instantaneous wall thickness of the ring during the rolling process. In Equations (3) and (4), the rolled ring is regarded as an equivalent ring with constant thickness H. In fact, due to the continuous feed of the mandrel, the thickness of the ring is varied along the circumference, and it is minimum at the exit gap between the mandrel and the main roll. In large-scale ring rolling, as the rolling time per revolution is long, the variation of thickness can be notable and affects the prediction for ring growth speed $v_{D}$ by using Equation (4). To take this variation into account, H is expressed as:

(5) $$H=\frac{\int_{t-T}^{t}\left(H_{0}-U\right)dt}{T}=H_{0}-\frac{\int_{t-T}^{t}Udt}{T}$$

where U is the displacement of the mandrel, T the time period to finish one revolution of the ring at every moment, and the derivative of U to T is the radial feed rate v. With Equations (4) and (5), the ring growth speed $v_{D}$ can be deduced as a function of radial feed rate v:

(6) $$v_{D}=\left[\frac{(D_{0}+d_{0})H_{0}}{2{\left(H_{0}-\frac{\int_{t-T}^{t}Udt}{T}\right)}^{2}}-1\right]\frac{\int_{t-T}^{t}vdt}{T}$$

**Figure 4:**
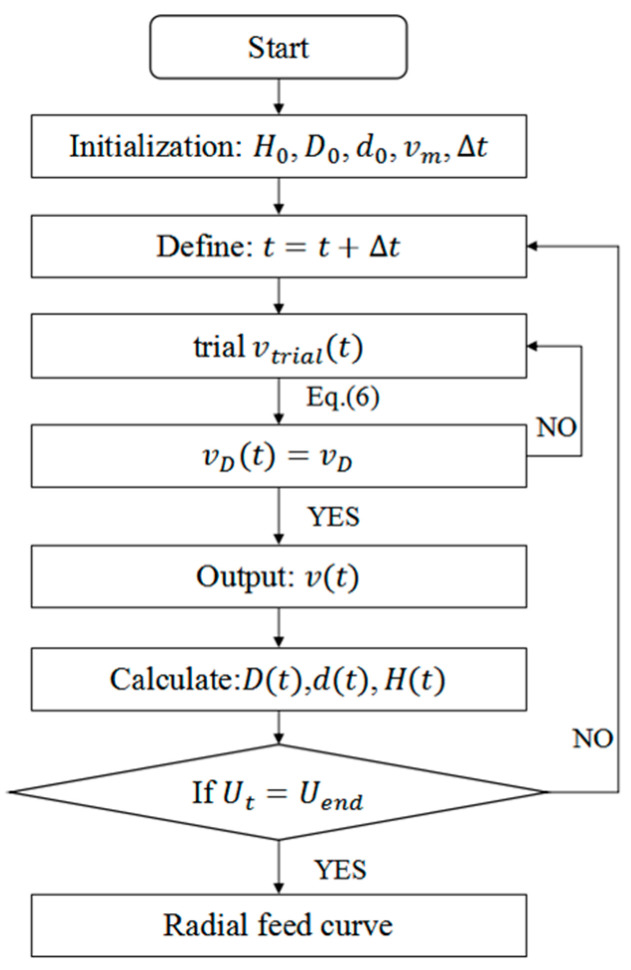
Flow chart of numerical computation for mathematical model.

Although the relationship between ring growth speed and radial feed rate is indicated by Equation (6), it is difficult to solve this function and work out the analytical solution. Here, a numerical computation method is presented to seek the solution to this mathematical model. In this method, the whole rolling process is divided into many small time increments. Due to its repeat step at every time increment, this method can be programmed with MATLAB 2022a (Natick, MA, United States) software and is computationally efficient. This procedure is calculated as follows and as shown in Figure 4: First, the initialization parameters including ring billet size $H_{0}$, $D_{0}$ and $d_{0}$, incremental steps ∆t and main roll line speed $v_{m}$ are set. Then, the current time *t* is determined. Next, the ring growth rate $v_{D}\left(t\right)$ is calculated for different feed rates $v_{trial}\left(t\right)$ according to Equation (6), and the feed rate $v\left(t\right)$ corresponding to the set ring growth rate $v_{D}$ is output according to the calculation result. Then, the radial feed amount $U_{t}$, the outer diameter *D*$\left(t\right)$, inner diameter *d*$\left(t\right)$ and wall thickness *H*$\left(t\right)$ of the ring at moment *t* are updated based on the output radial feed rate [11]. Finally, when $U_{t}$ reaches the set value $U_{end}$, the program ends. Through the mathematical model and numerical computation method, the feed curve of the mandrel and the growth speed of the ring can be calculated when one of them is given. Thus, radial feed curves of the initial, rapid and steady rolling stages are determined through the numerical computation for the mathematical model according to the constant ring growth speed, and the feed curve for the slow rolling stage takes an even deceleration at the final rate of the steady rolling stage.

### 3.2. Coordinated Radial Feed Curve Design 

The feed curves for the different rolling stages can be determined according to Section 3.1, but the quantitative division of the different stages needs further study. The total wall thickness reduction amount is composed of wall thickness reduction amounts in the initial rolling stage, rapid rolling stage, steady rolling stage and stable rolling stage. Hence, the quantitative division of different stages can be determined based on the percentages of the wall thickness reduction amount in different rolling stages. There are some restrictive conditions for the percentages of the wall thickness reduction amount in the rapid rolling stage (R) and slow rolling stage (S). 

To obtain the range of R and S, a three-dimensional thermodynamic coupling FE model for a 5 m 2219 aluminum alloy flat ring, which is illustrated in Figure 1, was established under the SIMUFACT 16.0 (Simufact Engineering, Hamburg, Germany) software platform. The main roll rotates the ring by friction, the mandrel moves according to the subsequently calculated feed curve, and the guide rolls and axial rolls are controlled according to the real-time outer diameter of the ring. Detailed parameters for FE modeling, including rolling speed, billet size and roll condition of the rolling process, are presented in Table 1, the type of mesh is hexahedral, and the constitutive model of the 2219 aluminum alloy used for the numerical simulation is obtained from the literature [26].

**Figure 5:**
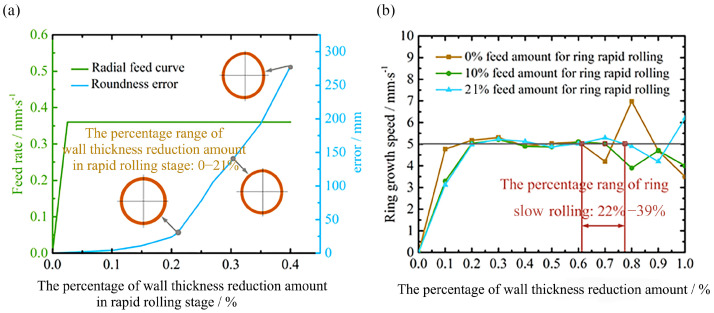
Range of rapid rolling stage (**a**) and slow rolling stage (**b**).

Figure 5a shows that roundness error obtained by the numerical simulation increased rapidly in the early rolling stage when the R exceeded 21%. A large roundness error is not beneficial for the stable growth of the ring in the stable rolling stage, so the R is in the range of 0 to 21%. In the range of R, S is further determined by analyzing the growth speed of the ring during the steady rolling phase. Figure 5b shows that the ring growth speed cannot be maintained constant when the percentage of thickness reduction is between 61% and 78%. This means that when the percentage of the wall thickness reduction amount reaches the range of 61% to 78%, the ring rolling should enter the slow rolling stage, so the S is in the range of 22% to 39%. 

**Figure 6:**
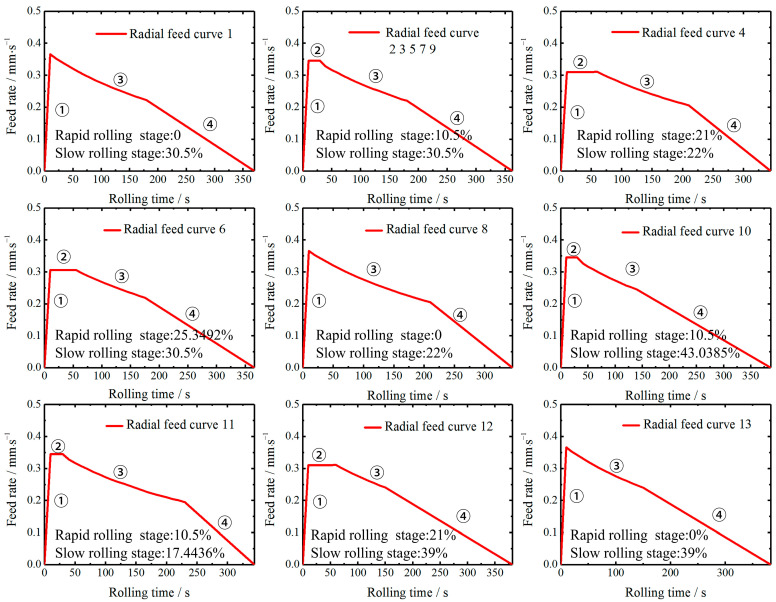
The radial feed curves designed by using CCD and mathematical model.

Based on the percentage range of wall thickness reduction in the rapid and slow rolling stages, the feed scheme is determined using the CCD method, and then a mathematical model established in Section 3.1 is applied to obtain the radial feed curves for the different schemes, as shown in Figure 6. To better compare the feed curves of different schemes, ①, ②, ③ and ④ are used to represent the initial, rapid, stable and slow rolling stages. Compared with the stage without ②, the feed rate with stage ② is generally lower. Combined with the previous analysis, the lower feed rate can reduce the rolling force, thus reducing *M* and *Mr* so that its roundness error, eccentricity and vibration phenomenon are improved. Additionally, the rapid stage can not only quickly increase the outer diameter of the ring early in the rolling but also effectively reduce *M* and *Mr* to control the rolling stability. Another feature is that if stage ④ has a larger percentage, the feed curve form of stages ③ and ④ is not too different. This indicates that the parameter of slow rolling stage percentage effectively determines whether the curve of stage ④ can play a role in controlling the rolling stability.

### 3.3. Stability Rolling Feed Curves

**Figure 7:**
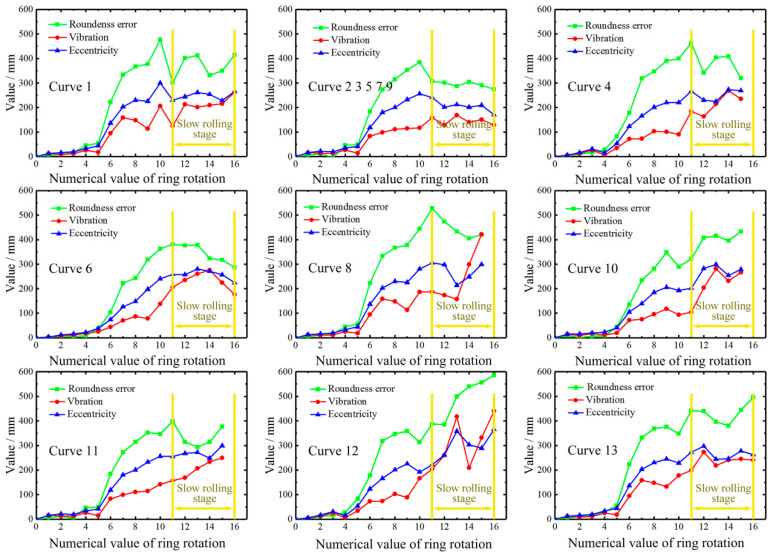
Roundness, eccentricity and vibration for different designed radial feed curves obtained by numerical simulation.

Figure 7 shows the maximum values of roundness error, eccentricity and vibration of different designed curves per revolution obtained by the numerical simulation. Among them, the roundness error is obtained by the least square circle method, the difference value between the *X*-axis of the ring in the relative coordinate system and the *X*-axis of the ring in the absolute coordinate system is defined as eccentricity, and the vibration floating distance is used as the result of vibration that is determined by calculating the difference value between maximum eccentricity and minimum eccentricity. Consistent with the previous analysis, the smaller ring diameter makes the ring rolling early instability shape lower, and a reduction in the radial feed rate in the slow rolling stage can effectively improve the increase in rolling instability. A comparison of the simulation results of different feed curves shows that among all the feed curves, feed curves 2, 3, 5, 6, 7 and 9 are effective in improving roundness error, eccentricity and vibration. Moreover, except for feed curves 10, 12 and 13, the slow rolling stages of all curves are effective in reducing roundness error, eccentricity and vibration. Combining the feed curves shown in Figure 6, it is found that feed curves 2, 3, 5, 6, 7 and 9, which effectively improve rolling stability, have a rapid rolling stage, while feed curves 10, 12 and 13 have a similar curve form of the stable rolling stage and slow rolling stage. Therefore, this shows that the rapid rolling stage has an indispensable role in improving rolling stability and that the slow stage has a reduced effective feed rate to effectively control rolling stability. In addition, this result further verifies that the coordination of different rolling stages can affect the maximum values of roundness error, eccentricity and vibration.

**Figure 8:**
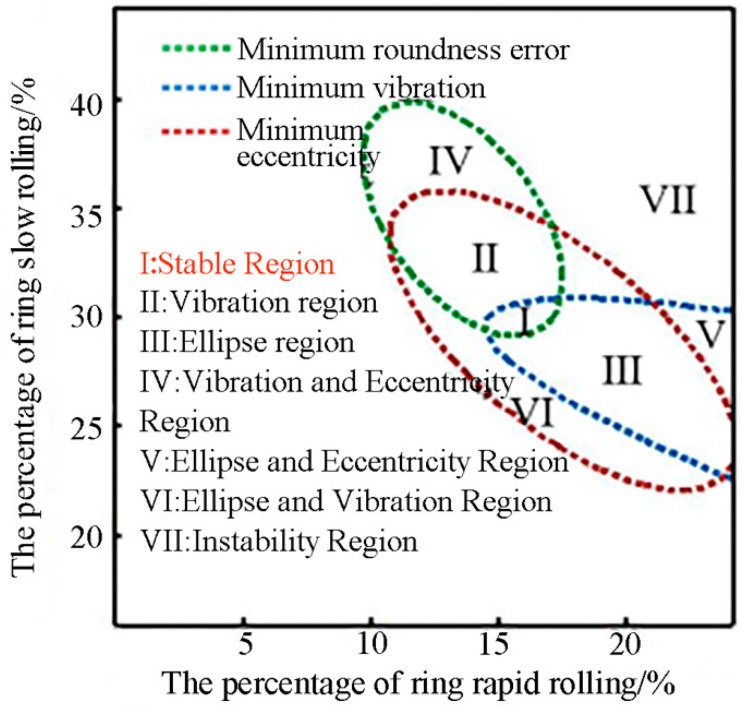
Rolling map with stable rolling region and rolling defects of different rolling regions.

Based on these results, the response surfaces model for maximum circularity error, maximum eccentricity and maximum vibration is established, where the variables are R and S. Furthermore, a rolling map shown in Figure 8 is established by superposing the minimum contour lines in the response surface of maximum roundness error (400 mm), maximum eccentricity (270 mm) and maximum vibration (170 mm), which can obtain the stable rolling region and reveal the different rolling defects of different rolling regions. This map can be divided into seven regions.

1. Region I is the stable rolling region. The roundness error, eccentricity and vibration in this region are all the minimum areas, so region I is determined by the minimum areas of roundness error, eccentricity and vibration.
2. Region II is the defect region with high vibration. In addition to vibration, roundness error and eccentricity in this region are the minimum areas, so the rolling process in this region has the phenomenon of high vibration. Region II is determined by the common area of roundness error and eccentricity.
3. Region III is the defect region with a serious ellipse. This region includes only the minimum areas of vibration and eccentricity, so the ring shape in the rolling process is more elliptical under the radial feed curve designed based on this region.
4. Region IV is the defect region with strong eccentricity and high vibration. Because this region is only within the minimum roundness error area, the rolling process in this region has the characteristics of strong eccentricity and high vibration.
5. Region V is the defect region with a serious ellipse and strong eccentricity. Due to this region being determined by the minimum of vibration, the roundness error and eccentricity value are not guaranteed to be minimum, so a serious ellipse and strong eccentricity are inevitable in this region.
6. Region VI is the defect region with a serious ellipse tendency and high vibration tendency. Only eccentricity in this region is within the minimum eccentricity area, so the ring in this region has a serious ellipse and high vibration.
7. Region VII is the instability rolling region. This region is outside of all minimum areas, which causes instability in the rolling process.

## 4. Verification of Feed Curve Designed by Stability Rolling Region

### 4.1. Verification of Stability Rolling Region

**Figure 9:**
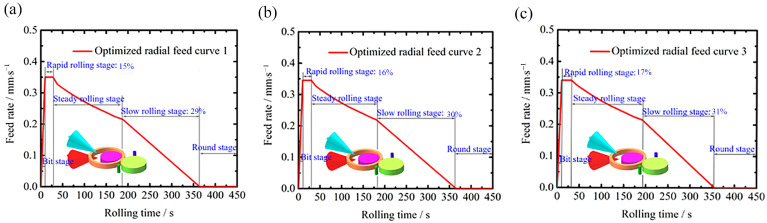
Optimized radial feed curve 1 (**a**), optimized radial feed curve 2 (**b**) and optimized radial feed curve 3 (**c**) designed based on stable rolling region.

**Figure 10:**
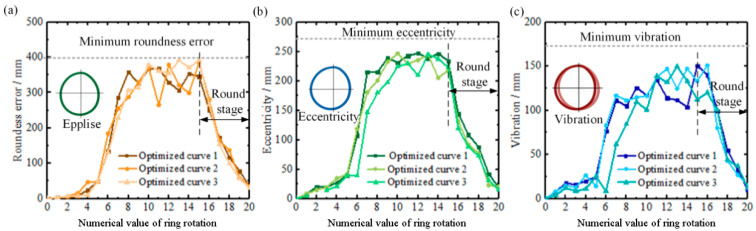
Maximum values of roundness error (**a**), vibration (**b**) and eccentricity (**c**) for different optimized radial feed curves.

To verify the feasibility of the stable rolling region, three radial feed curves are designed based on the stable rolling region. Furthermore, in order to improve roundness, the slow rolling stage is followed by a round stage in which the mandrel stops feeding but the ring is still rotating, as shown in Figure 9. Figure 10 describes the maximum values of roundness error, vibration and eccentricity per revolution for different optimized radial feed curves obtained by numerical simulation, and they are all below the minimum values that determine the stable rolling region, which demonstrates the feasibility of the designed radial curve based on the stable rolling region.

### 4.2. Verification of Feed Curve

**Figure 11:**
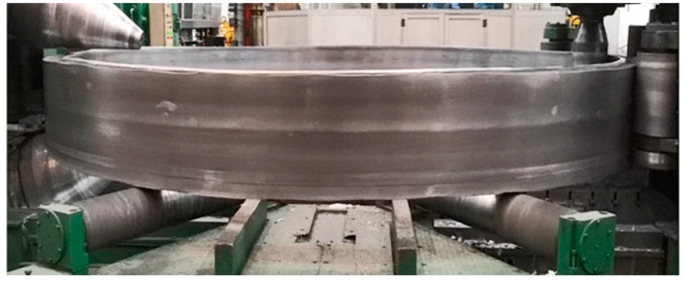
Production trial of large-scale flat ring.

In order to verify that the feed curve in the stable rolling region can meet the requirements, the optimized radial feed curve 3 was selected to carry out a production trial (Figure 11) and FE simulations for the ring rolling of 2219 aluminum alloy with a diameter of 5 m. Their ring billet size and other conditions are consistent with those shown in Table 1.

Based on the principle that three points determine a circle, the diameter of the rolled ring is calculated by measuring the distance between any three points selected on the outer circumference of the rolled ring. According to the above method, six groups of diameter values of the rolled ring are obtained, as shown in Table 2, which are used to construct the geometry of the rolled ring, thereby obtaining the value of roundness error, eccentricity and vibration for trial production. Compared with the trial production and numerical simulation results listed in Table 3, the results of roundness error, eccentricity and vibration are basically consistent. This indicates that it is reasonable to determine the feed curve through FE simulation.

The aim of controlling the rolling stability is to make the geometry of the rolled ring meet the requirements, which require a mechanical machining amount below 40 mm. The difference between the maximum and minimum values is the required machining amount of the rolled ring. As can be seen from Table 4, the machining amount required for both the production trials and the numerical simulations is below the maximum machining amount, thus satisfying the geometry requirements of the ring, which verifies the reliability of the radial feed curve in the stable rolling region.

## 5. Conclusions

This paper studies how to design the feed curve in a large-scale flat ring rolling process based on the characteristics of serious instability and large wall thickness difference. The conclusions are summarized as follows:

1. The roundness error, eccentricity and vibration of the rolled ring easily occur during large-scale ring rolling. The mechanism of roundness error and eccentricity is that the bending moment of the ring cross-section in the radial deformation zone is greater than the ultimate bending moment, which changes the radius and curvature of the ring in the circumferential direction. The vibration is caused by the loss of circularity of the ring, which prevents the guide roll from contacting the ring, thus generating a deflecting moment, which deflects the ring around the center of the mandrel.
2. The coordination of the different rolling stages influences the maximum value of the roundness error, eccentricity and vibration during rolling. The results of the analytical analysis and the numerical simulations are in agreement, and both indicate that the ring rolling stability is good at the beginning of rolling and that the outer diameter of the ring should be increased rapidly. Subsequently, the ring rolling stability increases rapidly such that the later stages of rolling need to be rolled slowly.
3. Based on the rolling map established by superposing the minimum contour lines in the maximum roundness error, eccentricity and vibration response surface, a stable rolling region is generated to determine the radial feed curve, and rolling defects of different rolling regions are revealed. Moreover, FE simulation results show that the values of roundness error, vibration and eccentricity per revolution for different optimized radial feed curves in the stable rolling region are all below the minimum values that determine the stable rolling region, which demonstrates the feasibility of the designed radial curve based on the stable rolling region.
4. A feed curve in the stable rolling region was selected to carry out a production trial and FE simulations for the ring rolling of 2219 aluminum alloy with a diameter of 5 m. Their results show that the roundness error, eccentricity and vibration obtained from the numerical simulation are basically the same as those from the production trial, and the mechanical machining amounts are all below the maximum value, indicating that the feed curve designed based on the stable rolling region can meet the geometry requirements of a large-scale ring.

## Figures and Tables

**Table 1:** Process parameters of the ring.

Parameters	Values
Main roll speed (rad/s)	1.0
Radial feed curve	Determined by ring growth speed of 5 mm/s
Axial feed amount (mm)	0
Friction coefficient	0.3
Initial billet temperature (°C)	500
Initial rolls temperature (°C)	150
Ring billet parameter (mm)	Φ3600 × Φ3000 × 500
Ring forging parameter (mm)	Φ5030 × Φ4820 × 500

**Table 2:** Measurement results obtained by production trial.

Implement	Production Trial					
	Group1	Group2	Group3	Group4	Group5	Group6
Diameter (mm)	5037	5028	5047	5066	5053	5037
Determination accuracy	0.0029%	−0.0019%	0.0022%	0.0028%	0.0014%	−0.0018%

**Table 3:** Comparison of production trial results and numerical simulation results.

Implement	Production Trial	Numerical Simulation	Relative Error
Roundness error (mm)	38	31	−18.42%
Eccentricity (mm)	25	22	−12.00%
Vibration (mm)	13	12	−7.69%

**Table 4:** Machining amount.

Object	Production Trial	Numerical Simulation	Requirement
Machining amount (mm)	38	31	≤40

## Data Availability

Not applicable.
